# Association between Parkinson's disease and proton pump inhibitors therapy in older people

**DOI:** 10.37796/2211-8039.1048

**Published:** 2020-09-01

**Authors:** Shih-Wei Lai, Kuan-Fu Liao, Cheng-Li Lin, Chih-Hsueh Lin

**Affiliations:** a College of Medicine, China Medical University, Taichung, Taiwan; b Department of Family Medicine, China Medical University Hospital, Taichung, Taiwan; c College of Medicine, Tzu Chi University, Hualien, Taiwan; d Division of Hepatogastroenterology, Department of Internal Medicine, Taichung Tzu Chi Hospital, Taichung, Taiwan; e Management Office for Health Data, China Medical University Hospital, Taichung, Taiwan

**Keywords:** proton pump inhibitors, older people, Parkinson's disease

## Abstract

**Objectives:**

The study was to explore the association between Parkinson's disease and proton pump inhibitors use.

**Methods:**

A population-based case-control study was conducted to analyze the 2000-2013 database of Taiwan National Health Insurance Program. In total, there were 4280 participants aged ≥65 years with newly diagnosed Parkinson's disease as the case group and 4280 sex- and age-matched participants without Parkinson's disease as the control group. Ever use of proton pump inhibitors was defined as participants who had at least a prescription for proton pump inhibitors before the index date. Never use of proton pump inhibitors was defined as participants who did not have a prescription for proton pump inhibitors before the index date. The odds ratio and 95% confidence interval were used to estimate the association between Parkinson's disease and proton pump inhibitors use by the logistic regression model.

**Results:**

A significant association was detected between Parkinson's disease and proton pump inhibitors use (odds ratio 1.15, 95% confidence interval 1.04-1.27).

**Conclusions:**

An association is found between Parkinson's disease and proton pump inhibitors use in older people. Other real-world data are required to confirm the clinical impact of proton pump inhibitors therapy on the risk of Parkinson's disease.

## 1. Introduction

Proton pump inhibitors are widely prescribed to treat acid-related disorders of upper gastrointestinal tract. The side effects resulting from long-term use of proton pump inhibitors are of greater concern.[1] One nested case-control study reported that no association could be detected between Alzheimer's Disease and proton pump inhibitors use.[2] In addition, previous studies demonstrated that proton pump inhibitors therapy were associated with increased risk of some comorbidities including pyogenic liver abscess (odds ratio 7.59, 95% confidence interval 5.05-11.4),[3] pulmonary tuberculosis (odds ratio 1.31, 95% confidence interval 1.22-1.41),[4] and chronic kidney disease (odds ratio 1.41, 95% confidence interval 1.34-1.48).[5] To date, little evidence is available on the association between Parkinson's disease and proton pump inhibitors therapy in Taiwan. Therefore, a case-control study was conducted to investigate this issue.

## 2. Methods

### 2.1. Data source and Study subjects

A population-based case-control study was per-formed to analyze the 2000-2013 database of Taiwan National Health Insurance Program. The Taiwan National Health Insurance Program is a whole-population health insurance system. The program Lunched on 1 March 1995 and it covered more than 99.7% of the 23 million persons living in Taiwan.[6-8] The details of the program have been found in previous studies.[9-14].

Participants aged ≥ 65 years with newly diagnosed Parkinson's disease were selected as the case group (based on International Classification of Dis-eases, Ninth Revision, Clinical Modification, ICD-9 code 332.0). Sex-matched, age-matched, and comorbidities-matched participants without Parkinson's disease were randomly selected as the control group. The index date was defined as the date of each case being diagnosed with Parkinson's disease. Participants who had a history of secondary Parkinsonism before the index date were excluded from the study. To decrease the latency bias, if the first-time prescription for proton pump inhibitors was found ≤12 months before the index date, these participants were excluded from the study.

The definition of proton pump inhibitors use was adapted from the previous studies.[15-20] Ever use of proton pump inhibitors was defined as participants who had at least a prescription for proton pump inhibitors before the index date. Never use of proton pump inhibitors was defined as participants who did not have a prescription for proton pump inhibitors before the index date.

### 2.2. Statistical analysis

The differences of demographic status, proton pump inhibitors use, and comorbidities between the case group and the control group were compared by using the chi-square test for categorized variables and by the *t*-test for continuous variables. The odds ratio and 95% confidence interval were used to assess the association between Parkinson's disease and proton pump inhibitors use by the logistic regression model. The duration-dependent effect of proton pump inhibitors use associated with the risk of Parkinson's disease was analyzed. All analyses were examined by using SAS statistical software (version 9.2; SAS Institute, Inc., Cary, North Carolina, USA). The results were considered statistically significant if two-tailed *P* values were <0.05.

## 3. Results

There were 4280 participants with Parkinson's disease in the case group and 4280 participants without Parkinson's disease in the control group. Both groups had equal distributions of sex, age, and comorbidities (Table 1). Nearly 50% of the study participants were males. The mean ages (standard deviation) were 76.5 (6.3) years in the case group and 76.4 (6.3) years in the control group, without statistic significance (*P* = 0.44 for *t*-test). The case group had a higher proportion of proton pump inhibitors use than the control group, with statistic significance (23.3% versus 20.9%, *P* = 0.008 for Chi-square test).

Because no variable was significantly associated with Parkinson's disease in the univariable model, the multivariable logistic regression model was not performed. A univariable logistic regression model demonstrated that a significant association was detected between Parkinson's disease and proton pump inhibitors use (odds ratio 1.15, 95% confidence interval 1.04-1.27; Table 2).

In further analysis, there was a significant association between Parkinson's disease and increase in duration for every one month of proton pump inhibitors use (odds ratio 1.02, 95% confidence interval 1.01-1.03; Table 3).

## 4. Discussion

The present study demonstrated that the odds of proton pump inhibitors therapy were 1.15 times higher in older people with Parkinson's disease versus those without Parkinson's disease. This finding was partially compatible with a case-control study in Denmark showing that proton pump inhibitors therapy before the diagnosis of Parkinson's disease for 5 years or longer remained to be associated with Parkinson's disease (odds ratio 1.23, 95% confidence interval 1.11-1.37).[21] This present study also demonstrated that that there was a duration-dependent manner of proton pump inhibitors use on the increased risk of Parkinson's disease. That is, the longer the proton pump inhibitors use, the greater the risk of Parkinson's disease.

To date, only few studies explored proton pump inhibitors therapy on the risk of Parkinson's disease. We were unable to compare them with each other. Although a significant association was detected between Parkinson's disease and proton pump inhibitors therapy, the causal relationship could not be determined by a case-control design. We suggest that other real-world data are required to illustrate the clinical impact of proton pump inhibitors therapy on the risk of Parkinson's disease.

Some limitations should be discussed. First, proton pump inhibitors and histamine-2 receptor antagonists had similar indications, but they could not be prescribed simultaneously under the Taiwan National Health Insurance Program. Therefore, histamine-2 receptor antagonists were not included in this present study for analysis. However, it indicates a future research direction for the association between Parkinson's disease and histamine-2 receptor antagonists therapy. Second, due to the limitation of the database used, smoking information was not available in the database. Therefore, chronic obstructive pulmonary disease was used for instead. Third, we found that 95.4% of Parkinson's disease cases and 97.6% of controls had ever used non-steroid anti-inflammatory drugs (Table not shown). The proportions were too high. It was not suitable to include non-steroid anti-inflammatory drugs for analysis even if non-steroid anti-inflammatory drugs could be associated with Parkinson's disease. Fourth, proton pump inhibitors could be prescribed only by the endoscopic diagnosis of upper gastro-intestinal diseases in Taiwan. It was less likely to use proton pump inhibitors to treat depression, which was often a prodromal symptom in Parkinson's disease. In spite of the above limitations, the present study has a novel concept that explores the association between Parkinson's dis-ease and proton pump inhibitors therapy.

We conclude that an association is found between Parkinson's disease and proton pump inhibitors use in older people. Other real-world data are required to confirm the clinical impact of proton pump inhibitors therapy on the risk of Parkinson's disease.

## Figures and Tables

**Table 1:** Characteristics of cases with Parkinson's disease and controls.

Variable	Controls N = 4280		Cases N = 4280		*P* value^a^
	n	(%)	n	(%)	
Sex					0.99
Female	2145	(50.1)	2145	(50.1)	
Male	2135	(49.9)	2135	(49.9)	
Age group (years)					0.99
65-74	1818	(42.5)	1818	(42.5)	
75-84	2057	(48.1)	2057	(48.1)	
≥85	405	(9.4)	405	(9.4)	
Age (years), mean ± standard deviation^b^	76.4 ± 6.3		76.5 ± 6.3		0.44
Ever use of proton pump inhibitors	895	(20.9)	997	(23.3)	0.008
Exposure duration of proton pump inhibitors (days), mean ± standard deviation^b^	136.6 ± 223.2		154.4 ± 220.1		0.08
Comorbidities					
Alcohol-related disease	90	(2.10)	90	(2.10)	0.99
Cerebrovascular disease	819	(19.1)	819	(19.1)	0.99
Chronic kidney disease	268	(6.26)	268	(6.26)	0.99
Chronic obstructive pulmonary disease	1804	(42.2)	1804	(42.2)	0.99
Diabetes mellitus	801	(18.7)	801	(18.7)	0.99
Hyperlipidemia	1384	(32.3)	1384	(32.3)	0.99
Hypertension	3549	(82.9)	3549	(82.9)	0.99

Data are revealed as the number of participants in each group, with percentages given in parentheses.

a Chi-square test.

b *t*-test comparing cases with Parkinson's disease and controls.

**Table 2:** Odds ratio and 95% confidence interval of Parkinson's disease associated with proton pump inhibitors use and comorbidities by logistical regression model.

Variable	OR	(95%CI)
Sex (male vs. female)	1.00	(0.92,1.09)
Age (every one year)	1.00	(0.99, 1.01)
Ever use of proton pump inhibitors (never use as a reference)	1.15	(1.04, 1.27)
Comorbidities (yes versus no)		
Alcohol-related disease	1.00	(0.74, 1.34)
Cerebrovascular disease	1.00	(0.90, 1.11)
Chronic kidney disease	1.00	(0.84, 1.19)
Chronic obstructive pulmonary disease	1.00	(0.92, 1.09)
Diabetes mellitus	1.00	(0.90, 1.12)
Hyperlipidemia	1.00	(0.91, 1.10)
Hypertension	1.00	(0.89, 1.12)

Because no variable was significantly associated with Parkinson's disease in the univariable model, the multivariable logistic regression model was not performed.

**Table 3:** Association of Parkinson's disease with cumulative duration of proton pump inhibitors use.

Variable	Case number/control number	Odds ratio (95% CI)
Never use of proton pump inhibitors as a reference	3283/3385	1.00 (reference)
Cumulative duration of proton pump inhibitors use (increase in duration for every one month)	997/895	1.02 (1.01, 1.03)

Because no variable was significantly associated with Parkinson's disease in the univariable model, the multivariable logistic regression model was not performed.
